# Benchmark Evaluation of True Single Molecular Sequencing to Determine Cystic Fibrosis Airway Microbiome Diversity

**DOI:** 10.3389/fmicb.2018.01069

**Published:** 2018-05-25

**Authors:** Andrea Hahn, Matthew L. Bendall, Keylie M. Gibson, Hollis Chaney, Iman Sami, Geovanny F. Perez, Anastassios C. Koumbourlis, Timothy A. McCaffrey, Robert J. Freishtat, Keith A. Crandall

**Affiliations:** ^1^Division of Infectious Diseases, Children’s National Health System, Washington, DC, United States; ^2^Department of Pediatrics, George Washington University School of Medicine and Health Sciences, Washington, DC, United States; ^3^Computational Biology Institute, Milken Institute School of Public Health, The George Washington University, Washington, DC, United States; ^4^Department of Microbiology, Immunology and Tropical Medicine, George Washington University School of Medicine and Health Sciences, Washington, DC, United States; ^5^Division of Pulmonary and Sleep Medicine, Children’s National Health System, Washington, DC, United States; ^6^Division of Genomic Medicine, The George Washington University, Washington, DC, United States; ^7^Department of Medicine, George Washington University School of Medicine and Health Sciences, Washington, DC, United States; ^8^Division of Emergency Medicine, Children’s National Health System, Washington, DC, United States

**Keywords:** cystic fibrosis, antibiotics, microbiome, metagenomics, true single molecule DNA sequencing

## Abstract

Cystic fibrosis (CF) is an autosomal recessive disease associated with recurrent lung infections that can lead to morbidity and mortality. The impact of antibiotics for treatment of acute pulmonary exacerbations on the CF airway microbiome remains unclear with prior studies giving conflicting results and being limited by their use of 16S ribosomal RNA sequencing. Our primary objective was to validate the use of true single molecular sequencing (tSMS) and PathoScope in the analysis of the CF airway microbiome. Three control samples were created with differing amounts of *Burkholderia cepacia*, *Pseudomonas aeruginosa*, and *Prevotella melaninogenica*, three common bacteria found in cystic fibrosis lungs. Paired sputa were also obtained from three study participants with CF before and >6 days after initiation of antibiotics. Antibiotic resistant *B. cepacia* and *P. aeruginosa* were identified in concurrently obtained respiratory cultures. Direct sequencing was performed using tSMS, and filtered reads were aligned to reference genomes from NCBI using PathoScope and Kraken and unique clade-specific marker genes using MetaPhlAn. A total of 180–518 K of 6–12 million filtered reads were aligned for each sample. Detection of known pathogens in control samples was most successful using PathoScope. In the CF sputa, alpha diversity measures varied based on the alignment method used, but similar trends were found between pre- and post-antibiotic samples. PathoScope outperformed Kraken and MetaPhlAn in our validation study of artificial bacterial community controls and also has advantages over Kraken and MetaPhlAn of being able to determine bacterial strains and the presence of fungal organisms. PathoScope can be confidently used when evaluating metagenomic data to determine CF airway microbiome diversity.

## Introduction

Cystic fibrosis (CF) is an autosomal recessive disease that affects more than 30,000 people in the United States (MacKenzie et al., 2014). Patients suffer from recurrent and chronic pulmonary infections that are strongly associated with morbidity and mortality (Ramsey, 1996). Recent use of culture-independent next generation sequencing (NGS) has identified novel and diverse communities of microbes in the CF airway, leading to an alteration in the traditional understanding of the role of infection in progressive lung disease (Huang and LiPuma, 2016). Decreasing microbial diversity is clearly associated with the presence of *Pseudomonas aeruginosa* and increasing age (Cox et al., 2010; Klepac-Ceraj et al., 2010; Boutin et al., 2015; Coburn et al., 2015). Cross-sectional studies have shown a difference in the structure and composition of airway microbiota between stable patients and those experiencing severe pulmonary decline, with decreased diversity in those with more advanced disease (Coburn et al., 2015; Flight et al., 2015; Bacci et al., 2016).

Antibiotic use has dramatically improved the longevity of children with CF and are likely responsible for perturbations of the airway microbiome (VanDevanter and LiPuma, 2012). However, the impact of antibiotics for treatment of acute pulmonary exacerbations on the CF airway microbiome remains unclear. Some studies have found that antibiotics do not lead to significant changes within the airway microbiome. Specifically, Fodor et al. (2012) found small decreases in bacterial richness but minimal changes in the overall community structure. Price et al. (2013) also followed CF patients through acute pulmonary exacerbations and found that the total and relative abundance of bacterial genera were stable during the exacerbation and after antibiotic treatment. Cuthbertson et al. (2016) also found that certain core microbiota remained resilient, regardless of exacerbation or antibiotic treatment state, but that rare species had much greater variability over time. However, other studies have reported that microbial diversity and the proportion of pathogenic bacteria decreased following antibiotic treatment. Zemanick et al. (2013) and Smith et al. (2014) found a decrease in microbial diversity early in the treatment course (around 72 h), which was also associated with a high relative abundance of *P. aeruginosa*. Later in the antibiotic treatment course (>7 days), the diversity appeared to return (Smith et al., 2014).

Longitudinal studies in patients with CF have shown long-term antibiotic use has also been associated with decreasing microbial diversity over time (Klepac-Ceraj et al., 2010; Zhao et al., 2012). However, increased antibiotic exposure is also confounded by age and declining lung function, making the establishment of a direct link between antibiotic use and microbial diversity difficult (Zhao et al., 2012).

Most of these prior studies investigating the impact of antibiotics on changes in the CF airway microbiome used 16S ribosomal RNA (rRNA) sequencing. This may be a limitation, as this approach requires PCR amplification that may suffer from primer bias in terms of accurate assessment of relative frequencies of bacterial taxa. There may also be a lack of differentiation amongst species with highly similar 16S sequences (Hilton et al., 2016). Metagenomic studies of the CF airway can give an unbiased look at the microbiome, and can also provide details on pathogen strain types (Feigelman et al., 2017). Furthermore, metagenomics studies could be used investigate the presence of antibiotic resistance genes or other fitness-conferring mutations (Feigelman et al., 2017). Our primary objective with this study was to validate the use of a metagenomic sequencing approach using true single molecular sequencing (tSMS, SeqLL Inc.) technology and the PathoScope computational framework (Francis et al., 2013; Hong et al., 2014) in CF airway samples.

### Materials and Methods

### Creation of Control Samples for Method Validation

Approximately 5 μg of dehydrated genomic bacterial DNA for *P. aeruginosa* (ATCC^®^ 47085D-5, strain PAO1-LAC), *B. cepacia* (ATCC^®^ 25416D-5), and *Prevotella melaninogenica* (ATCC^®^ 25845D-5) were obtained from ATCC (Manassas, VA, United States). To re-suspend the genomic DNA, 60 μL of molecular grade water were added to each sample. The samples were centrifuged (2000 *g* × 10 s) and incubated while continuously rocking overnight at 4°C. They were then incubated at 65°C for 1 h and then measured using a NanoDrop^TM^ spectrophotometer. Measured DNA concentrations were 194.2 ng/μL for *P. aeruginosa*, 187.8 ng/μL for *B. cepacia*, and 147.8 ng/μL for *P. melaninogenica*. Different proportions of these bacterial DNA were mixed together to create artificial community controls. Each 100 ng of Control A contained 20.7 ng of *P. aeruginosa*, 40 ng of *B. cepacia*, and 39.3 ng of *P. melaninogenica.* Control B contained 36.7 ng of *P. aeruginosa*, 35.4 ng of *B. cepacia*, and 35.4 ng of *P. melaninogenica* per 100 ng. Control C contained 47.5 ng of *P. aeruginosa*, 34.4 ng of *B. cepacia*, and 18.1 ng of *P. melaninogenica* per 100 ng. These mixtures were then frozen at -80°C until sequencing was performed.

### Patients and Sample Collection

The creation of a bio- and data repository was approved 08DEC2015 by the Institutional Review Board (Pro6781) at Children’s National Health System. Study subjects were consented for participation in the study prior to respiratory sample collection and extraction of data from electronic medical records. Paired sputa were obtained from three participants with documented antibiotic resistance for this study. Patient demographics and sample details are reported in **Table 1**.

**Table 1:** Study subject demographics, cultured bacteria, and treatment antibiotics.

Subject	Sex	Age (years)	Race/ethnicity	CFTR genotype	Time of collection	Culture results	Treatment antibiotics
S1A	F	23	White, non-Hispanic	1548delG	Day 0, pre-antibiotic	3+*Burkholderia cepacia*, 2+normal respiratory flora	Meropenem, ceftazidime, levofloxacin, trimethoprim/sulfamethoxazole
S1B					Day 6, post-antibiotic	3+*Burkholderia cepacia*, 3+normal respiratory flora	
S2A	F	21	White, Hispanic	F508del/3876delA	Day 0, pre-antibiotic	2+rough *Pseudomonas aeruginosa*, 2+rough *P. aeruginosa*, 2+mucoid *P. aeruginosa*, 2+mucoid *P. aeruginosa*, 4+normal respiratory flora	Ciprofloxacin
S2B					Day 14, post-antibiotic	2+mucoid *P. aeruginosa*, 2+normal respiratory flora	
S3A	M	7	White, Hispanic	F508del/F508del	Day 0, pre-antibiotic	3+rough *P. aeruginosa*, sparse rough *P. aeruginosa*, sparse mucoid *P. aeruginosa*, 2+normal respiratory flora	Ceftazidime, tobramycin
S3B					Day 6, post-antibiotic	4+rough *P. aeruginosa*, 2+rough *P. aeruginosa*	


### Respiratory Sample Collection and Processing

Per the biorepository protocol, spontaneously expectorated sputum samples obtained for clinical care were collected from the microbiology laboratory within 24 h of the patient’s clinical visit. Sputum samples were stored in a 4°C refrigerator prior to processing. For processing, sputum samples were mixed with Sputasol (dithiothreitol, Fisher Healthcare, Houston TX, United States), vortexed, and placed in a 37°C heated bead bath to homogenize the sample. The homogenized sputum was pelleted through centrifugation (12,000 *g* × 10 min). Supernatants were removed and bacterial pellets were frozen at -80°C until they underwent DNA extraction.

### Respiratory Culture Results

Clinical culture results within the electronic medical record were used to identify the pathogen and MICs for various antibiotics. The clinical microbiology laboratory uses MicroScan (Beckman Coulter, Brea, CA, United States) to determine identification and susceptibility of bacterial pathogens grown in culture and has an internally validated protocol it uses for mucoid *Pseudomonas aeruginosa* (Zimmer et al., 2004).

### DNA Extraction

Pelleted bacterial cells were rapidly thawed and mixed with 1 mL of sterile phosphate buffered saline (PBS). Bacterial DNA was extracted using a QIAamp DNA Microbiome kit (Qiagen, Valencia, CA, United States), following the protocol as outlined by the company. This kit was chosen as it has been reported to increase the ratio of bacterial to human DNA extracted (Qiagen, 2016).

### Metagenomic NGS

Metagenomic NGS was performed using tSMS (SeqLL Inc., Woburn, MA, United States). A starting amount of at least 300 ng of DNA (range 300–3000 ng) was used. Samples were prepared by first shearing to 100–200 nucleotides to create the appropriate sized fragments. This was followed by poly-A tailing and 3′ end blocking for capture on the flow cell surface. Two sequencing runs were performed, with the first loading 11.5 ng of DNA per sample and the second loading 16 ng of DNA per sample. The samples were then sequenced using 18 channels of a flow cell (two channels per sample). One channel was used for the run reference oligo. The instrument was operated at 550 field of view depth.

### Bioinformatic and Statistical Analysis

Raw reads were filtered by SeqLL to those with a quality reference score at or above 4.4/5.0 and with a length cutoff of 24 bases. The quality score considers the length of the aligned read, number of matches, and number of errors when it is normalized to the length of each read. The formula used is score = (number of matches^∗^5-number of mismatches^∗^4)/read length (Kapranov et al., 2010). Filtered reads per channel ranged from between 7.3 million to 13.3 million. The internal control oligo generated an observed mean length that indicated operational performance that was consistent with optimal system operational specifications.

FASTQ files containing filtered reads were aligned to reference genomes using PathoScope (Hong et al., 2014), Kraken (Davis et al., 2013), and MetaPhlAn (Segata et al., 2012). PathoScope and Kraken attempt to remove human sequences before aligning to microbial reference genomes. The reference database for PathoScope was created using sequences identified in the National Center for Biotechnology Information (NCBI) Archaea, Bacteria, Virus, and Fungal reference and representative genome database, which contains at least one genome for each species in the Entrez genome collection that has assembly data. To this we added all complete genome assemblies for *P. aeruginosa*, *B. cepacia*, and *Burkholderia cenocepacia*, thus enabling strain-specific identification of these species. The Kraken reference database also included NCBI bacterial and viral reference genomes. PathoScope and Kraken were run using the Colonial One High-Performance Computing Cluster at GWU. Reference contigs with unusually high read counts were screened against the nt database using BLAST; contigs determined to be contaminants (e.g., human sequences) were removed before analysis. MetaPhlAn was run using bioBakery v1.7, a virtual environment operated by the Huttenhower Lab (bioBakery, 2017).

Alpha diversity was measured as the number of species identified, the Shannon-Weiner Index, and the Simpson’s Reciprocal Index. The Shannon-Weiner Index was calculated in Excel (Microsoft, Redmond, WA, United States) using the equation $-\Sigma \left[In\,\;\left(n_{i}/\Sigma N\right)\right]$. The Simpson’s Reciprocal Index was calculated using the equation $1/\Sigma \left[n_{i}^{*}\,\;(n_{i}-1)/\left(\Sigma N^{*}\left(\Sigma N-1\right)\right)\right]$. Continuous variables were compared using t-test, while percentages of relative taxonomic abundance were compared using linear regression or McNemar’s test for correlated proportions. Taxonomy and metadata files were imported into *phyloseq* (McMurdie and Holmes, 2013) within R. Geometric means were used to estimate size factor and dispersion estimates, and differentially abundant species were identified using log2 fold change (adjusted *p*-value < 0.05) as implemented in *DESeq2* (Love et al., 2014). PERMANOVA was also calculated to measure the differences in overall bacterial distribution using the *adonis* function of *vegan* in R (Oksanen et al., 2017). Lastly, principle coordinates analysis (PCoA) plots were generated using Bray–Curtis distance matrices with log transformed counts to visualize differences between computational frameworks.

## Results

### Control Sample Comparison

We analyzed the tSMS generated metagenomics data with PathoScope resulting in the identification of a range of 33–73 bacterial/viral strains per control sample. The Kraken analysis of the same data resulted in the identification of a range of 442–518 bacterial/viral strains per sample, and the MetaPhlAn analysis resulted in the identification of a range of 55–76 bacterial/viral strains per sample.

When looking individually at the proportions between each comparison, it can be appreciated that PathoScope was more representative of the true amounts of bacteria used to create the artificial communities than Kraken or MetaPhlAn (**Table 2**). These differences in proportions were measured using linear regression. PathoScope had higher *r*^2^-values than Kraken in all comparisons, and had higher *r*^2^-values than MetaPhlAn two out of three times. In fact, PathoScope was significantly similar to the added proportions in Control A (*p* = 0.041), and approached significance in Control B (*p* = 0.071).

**Table 2:** Expected and obtained proportions of artificial communities.

	*Burkholderia cepacia* complex	*Pseudomonas aeruginosa*	*Prevotella melaninogenica*	Other	*r*^2^	*p*-value^∗^
**Control A**						
% added	40.0	20.7	39.3	0	NA	NA
% detected with PathoScope	45.3	22.9	31.8	0.04	0.919	**0.041**
% detected with Kraken	11.8	27.3	46.3	14.6	0.160	0.600
% detected with MetaPhlAn	18.7	59.7	19.3	2.1	0.032	0.822
**Control B**						
% added	35.4	36.7	27.9	0	NA	NA
% detected with PathoScope	30.7	32.4	37.0	0.001	0.863	0.071
% detected with Kraken	7.1	34.2	48.2	10.4	0.123	0.649
% detected with MetaPhlAn	10.6	62.9	24.9	1.5	0.402	0.366
**Control C**						
% added	34.4	47.5	18.1	0	NA	NA
% detected with PathoScope	32.9	30.4	36.7	0	0.506	0.288
% detected with Kraken	7.7	32.3	49.1	10.8	0.025	0.841
% detected with MetaPhlAn	11.1	63.3	23.1	2.3	0.623	0.211


*^∗^Linear regression, two-sided p-value. Bold indicates *p* < 0.05.*

### Cystic Fibrosis Sample Comparison

#### Metagenomic Sequencing

Six sputum samples from three study subjects who experienced an acute pulmonary exacerbation and whose respiratory cultures grew antibiotic-resistant bacteria were sequenced (see **Table 1**). Across all six samples, a total of 36 million sequencing reads passed quality control filters (6–12 M reads per sample). The filtered reads were assigned taxonomic labels using three metagenomic taxonomic classifiers: PathoScope, Kraken, and MetaPhlAn. PathoScope and Kraken align against whole reference genomes, while MetaPhlAn uses a reference set of clade-specific marker genes. With PathoScope, 3.6% (range 2.7–4.4%) of the total reads were initially aligned to genomes within the bacterial and viral reference database. Of these reads, 66% (range 48–87%) of the reads were removed as they aligned to human genome sequences during the filtering process. Ultimately, 1.3% (range 0.5–1.8%) of the total sequences were aligned to bacterial and viral reference genomes. With Kraken, 13% (range 10–16%) of the reads were classified. After filters were applied for human reads, 2.4% (range 0.4–4.1%) of the classified reads were identified as microbial. Of the classified reads, 2.3% (range 0.3–4%) were identified as bacterial and 0.002% (0.001–0.003%) were identified as viral. MetaPhlAn output was reported as relative abundance of microbial species after filtering, so the above determinations of aligned/classified and human filtering was not possible. However, of the 100% microbial reads reported per sample, 54% (range 16–77%) were identified as bacterial and 40% (range 20–59%) were identified as viral. Kraken identified the most distinct bacterial and viral (including bacteriophage) species (*n* = 516), while MetaPhlAn identified the next most (*n* = 202) followed by PathoScope (*n* = 91). PathoScope was also able to provide strain level information, identifying 283 strains total; two strains of *B. cepacia*, 9 strains of *B. cenocepacia*, and 109 strains of *P. aeruginosa* were detected within the six sputum samples.

**FIGURE 1:**
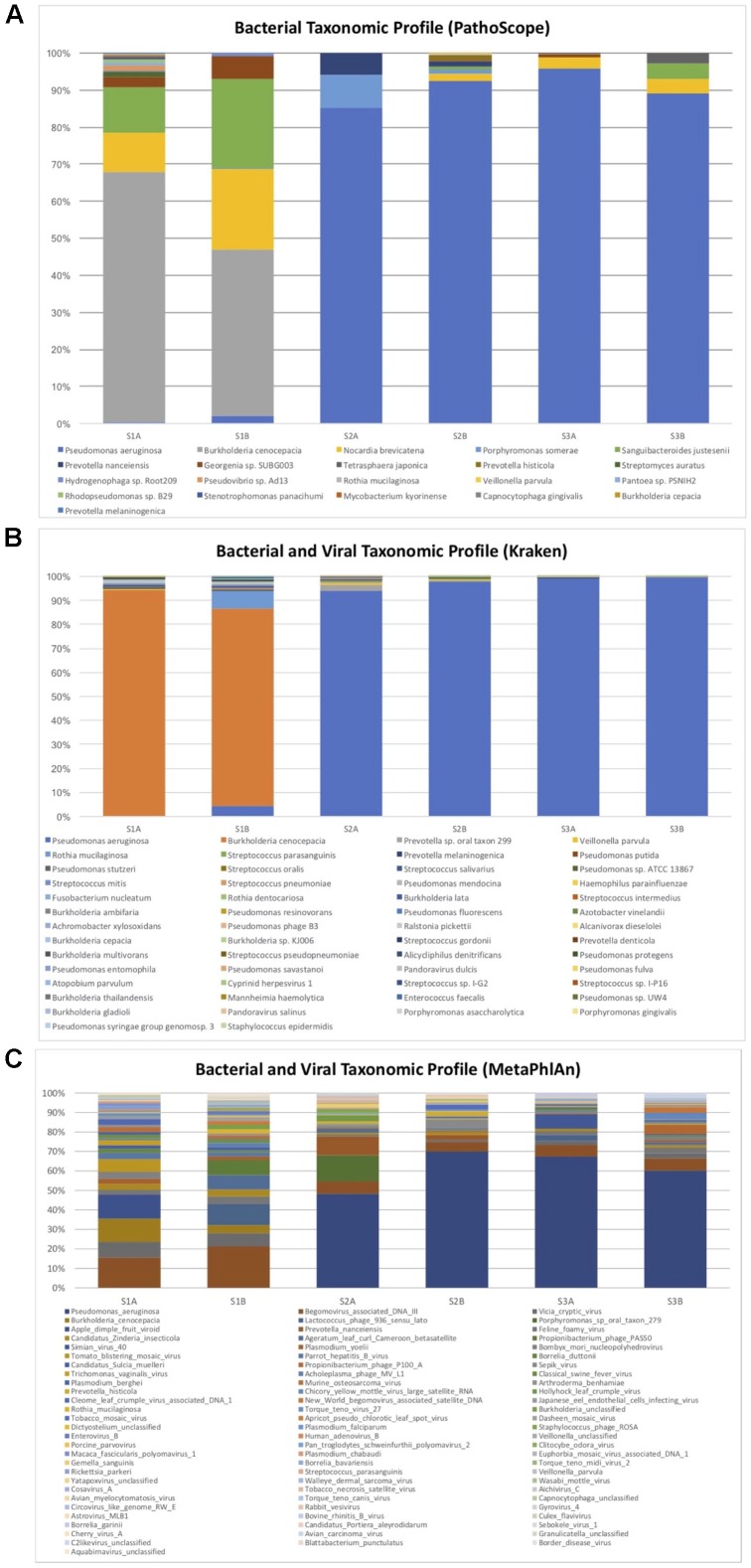
Bacterial and viral taxonomic profile of pre- and post-antibiotic sputum samples in three subjects with cystic fibrosis who grew antibiotic resistant bacteria. Only bacterial and viral species with a minimum total observation count of 0.01% of total reads are shown for PathoScope **(A)** and Kraken **(B)**. Only bacterial and viral species with a minimum total observation count of 0.1% of total reads are shown for MetaPhlAn **(C)**. *Burkholderia cepacia* was the pathogen identified in culture for subject S1, but the majority of reads were attributed to *Burkholderia cenocepacia. Pseudomonas aeruginosa* was identified as the predominant bacteria for subjects S2 and S3, and was also identified in corresponding respiratory cultures.

With PathoScope, fifty-one bacteria contributed to more than 0.01% of aligned reads per sample, and only 22 bacteria contributed to more than 0.01% of all total aligned reads. The bacterial taxonomic profile of each of the samples is showed over 83% of the total reads were aligned to *P. aeruginosa* and 4.7% aligned to *B. cenocepacia* (**Figure 1A**). The remaining reads that attributed to more than one percent of total aligned bacteria were *Nocardia brevicatena* (3.1%), *Porphyromonas somerae* (2.4%), *Sanguibacteroides justesenii* (2.2%), and *Prevotella nanceiensis* (1.7%). No viruses were detected with over 0.01% contribution to all total aligned reads.

With Kraken, 130 species contributed to at least 0.01% of the aligned reads per sample, and 54 species contributed to 0.01% of all aligned reads. The bacterial and viral taxonomic profile of each of the samples showed over 93% of the total reads aligned to *P. aeruginosa*, while 3.5% of total aligned reads were *B. cenocepacia* (**Figure 1B**). The remaining identified bacteria that contributed to more than 0.1% of total aligned reads were *Prevotella* sp. oral taxon 299 (0.7%), *Veillonella parvula* (0.3%), *Rothia mucilaginosa* (0.2%), *Streptococcus parasanguinis* (0.2%), and *Prevotella melaninogenica* (0.1%). Other bacteria identified within the *B. cepacia* complex include *B. ambifaria* (0.03%), *B. lata* (0.03%), *B. cepacia* (0.02%), and *B. multivorans* (0.01%). *Pseudomonas* phage B3 was detected with 0.02% contribution to all total aligned reads.

With MetaPhlAn, 201 species contributed to at least 0.01% of the aligned reads per sample, and 175 species contributed to 0.01% of all aligned reads. One hundred sixty three species contributed to at least 0.1% of aligned reads per sample, while 82 contributed to 0.1% of all aligned reads. The bacterial and viral taxonomic profile of each of the samples showed over 38% of the total reads aligned to *P. aeruginosa*, while 2.6% of reads aligned to *B. cenocepacia* (**Figure 1C**). *Porphyromonas* and *Prevotella* species, commonly identified in the CF lung, were identified at more than 1% of total aligned reads. The majority of other high contributors to the community identified were viruses and phages.

When comparing diversity indices at the species level there were no significant differences identified by the Shannon-Weiner index or the Simpson’s reciprocal index across all computational platforms (**Table 3**). Significant differences were seen with a decreased species count from pre- to post-antibiotics using Kraken (*p* = 0.023), and a decrease in the proportion of cultured bacteria using PathoScope (*p* = 0.016). We also measured bacterial distributions pre- versus post-antibiotics using Bray–Curtis distance matrices by PERMANOVA. There was no significant difference detected with either platform (Kraken *p* = 0.05, PathoScope *p* = 0.6, and MetaPhlAn *p* > 0.999).

**Table 3:** Alpha diversity indices and percentage of reads attributed to the cultured pathogen at the species level.

	Pre-antibiotic			Post-antibiotic			*P*-value^†^		
			
	PS	K	MPA	PS	K	MPA	PS	K	MPA
No. of species (mean ± SD)	27 ± 32	196 ± 44	72 ± 14	12 ± 4	139 ± 44	54 ± 9	0.534	**0.023**	0.089
Shannon-Weiner index (mean ± SD)	0.680 ± 0.465	0.341 ± 0.172	2.45 ± 0.525	0.747 ± 0.430	0.425 ± 0.429	2.28 ± 0.570	0.588	0.738	0.577
Simpson’s reciprocal index (mean ± SD)	1.525 ± 0.438	1.105 ± 0.059	8.045 ± 6.314	1.880 ± 0.944	1.200 ± 0.241	6.433 ± 5.421	0.450	0.586	0.271
Percentage cultured pathogen^∗^ (mean %)	82.6%	95.9%	39.9%	75.5%	93.0%	42.6%	**0.016**	0.250	0.250


**^∗^The cultured pathogen for subject S1 was considered all Burkholderia species present within Burkholderia cepacia complex (B. ambifaria, B. cepacia, B. cenocepacia, B. lata, and B. multivorans). The cultured pathogen for subjects S2 and S3 was Pseudomonas aeruginosa. ^†^Two-sided t test, paired samples for continuous variables; McNemar’s test, two-sided for proportions*. Bold indicates *p* < 0.05.*

**FIGURE 2:**
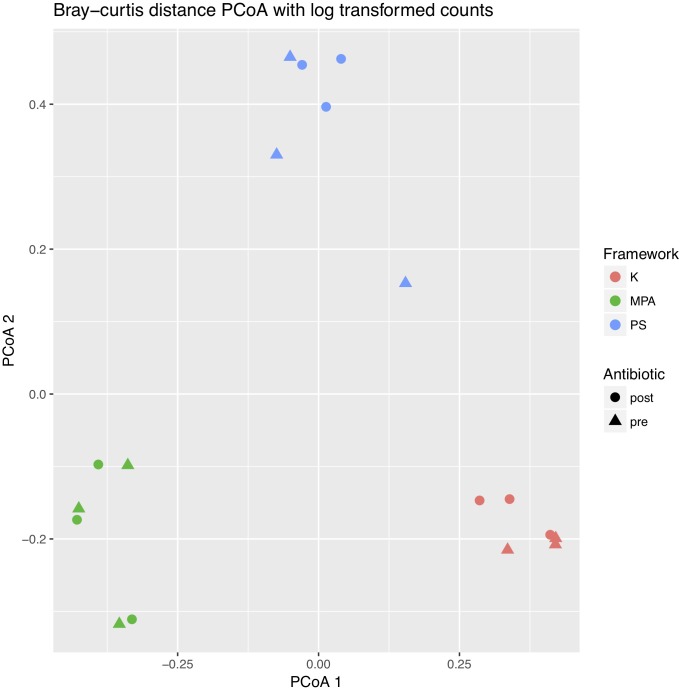
Two-dimensional principle coordinates analysis (PCoA) plot of pre- and post-antibiotic samples analyzed across computational frameworks. The PCoA was created using Bray–Curtis distance matrices with log transformed counts. Differences in sample types are shown by different shapes, while differences in computational framework are shown by different colors. K, Kraken, MPA, MetaPhlAn, PS, PathoScope.

Next, to better evaluate potential differences by computational framework, we performed a Bray–Curtis PCoA plot using log transformed counts (**Figure 2**). PERMANOVA again revealed no difference by antibiotic timing (*p* = 0.993), but did detect a significant difference by computational framework (*p* = 0.001). The subsequent permutation test for homogeneity of multivariate dispersions was not significant (*p* = 0.989).

**FIGURE 3:**
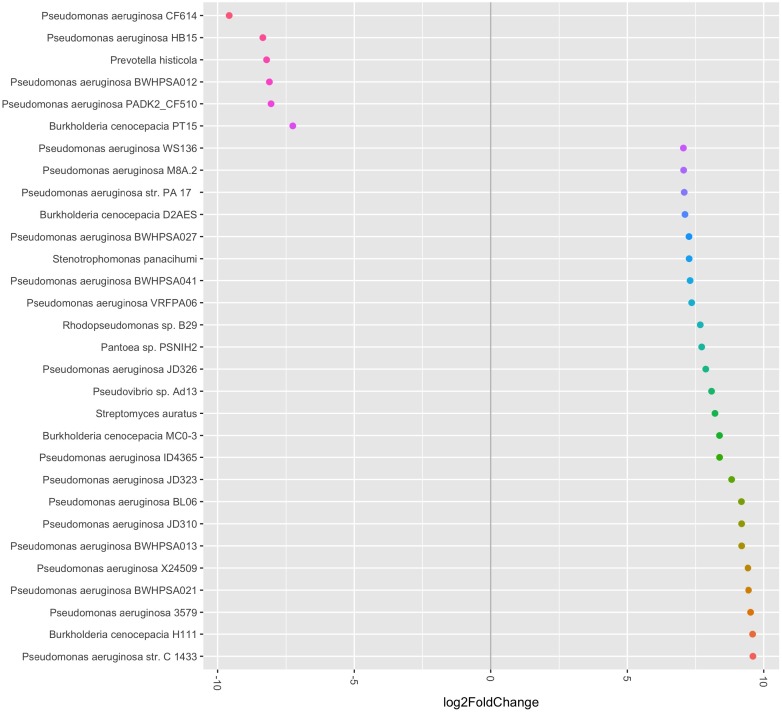
Relative abundance of bacterial species and strains pre- and post-antibiotic treatment. The bacterial species and strains plotted on the left side of the graph were more abundant in the post-antibiotic samples, while the bacterial species and strains plotted on the right side of the graph were more abundant in the pre-antibiotic samples. All fold-changes are significant at *p* < 0.05.

When evaluating the PathoScope data at the strain level, there were again no significant differences noted in alpha diversity pre- and post-antibiotic treatment. The pre- and post-antibiotic Shannon-Weiner diversity was 1.798 (0.433) vs. 1.464 (0.083), respectively (*p* = 0.310). The pre- and post-antibiotic Simpson’s reciprocal index was 4.256 (1.531) vs. 3.052 (0.274), respectively (*p* = 0.318). There was also no significant difference identified by PERMANOVA (*p* = 0.9). However, using *phyloseq* and *DESeq2* to evaluate strain specific data generated in PathoScope, we found several significant differences pre- and post-antibiotics (see **Figure 3**). *Prevotella histicola*, one *B. cenocepacia* strain, and four *P. aeruginosa* strains were more abundant in the post-antibiotic samples.

Kraken and MetaPhlAn focus solely on bacterial and viral species identification and do not identify fungal sequences from metagenomic data. PathoScope allows for metagenomics data to be aligned to fungal reference genomes. Ninety-two fungal species were identified that contributed to at least >0.1% of total fungal reads amongst all six samples. Approximately 4 and 1.4% of total fungal reads were assigned *Aspergillus* and *Candida* species, respectively, which are both known fungal pathogens in CF (Delhaes et al., 2012; Willger et al., 2014).

## Discussion

16S rRNA sequencing has traditionally been used to describe the airway microbiome in cystic fibrosis patients (Harris et al., 2007; Tunney et al., 2008; Fodor et al., 2012; Zhao et al., 2012; Zemanick et al., 2013; Carmody et al., 2013; Lim et al., 2014). There are many reasons for this, but part of it has to do with human DNA contamination within respiratory samples that makes sample processing complex (Lim et al., 2014). By limiting to 16S rRNA sequencing, however, the resolution for microbiome characterization is limited. If we do not identify bacteria to their species level, we may not discover the differing roles of organisms such as *Prevotella* based on their species or strain (Zemanick et al., 2013; Sherrard et al., 2014, 2016). Furthermore, metagenomic sequencing can also identify viruses, including bacteriophages, which can harbor antibiotic resistance genes or increase bacterial growth and virulence (Willner and Furlan, 2010; Willner et al., 2012). Thus, we sought to establish a technique of performing metagenomic sequencing of the cystic fibrosis airway microbiome using tSMS and PathoScope. By eliminating artificial bias, tSMS may has been successfully used in other areas but has not previously been used to study the CF airway microbiome (Orlando et al., 2011; Ginolhac et al., 2012; SEQLL, 2016). PathoScope, which also has not previously been used to study the CF lung, has successfully been used to filter out human reads and accurately identify pathogens within clinical samples (Francis et al., 2013; Hong et al., 2014; Byrd et al., 2014; Pérez-Losada et al., 2015). We compared our PathoScope results to results generated using Kraken (Davis et al., 2013) and MetaPhlAn (Segata et al., 2012).

The use of different NGS platforms and bioinformatic analysis techniques can impact both pathogen identification and diversity measures (Hahn et al., 2016). Our initial study of three control samples was encouraging that this combination of techniques would be successful in accurately detecting *B. cepacia* and *P. aeruginosa*. Control C showed much more variability than Controls A and B. This may be due to pipetting errors as this control sample was created last, or due to errors in sequencing as there were a large number of bacterial strains detected in this sample and almost 0.2% of taxonomic ID calls were for bacteria not added to the sample.

Our results demonstrate the ability to detect *P. aeruginosa* effectively using our metagenomic approach, which is a very important pathogen in CF (Harris et al., 2007; Carmody et al., 2013; Zemanick et al., 2013; Smith et al., 2014). This species grew in the respiratory cultures of two out of three study participants and was easily identified in those four samples. It was also detected to be part of the airway microbiome of the third subject, and the total number of reads aligned to *P. aeruginosa* was more than 47%. We were also able to easily identify *B. cenocepacia* and *B. cepacia*, which are also important pathogens within the CF airway (Fodor et al., 2012). It should be noted that *Burkholderia cepacia* complex includes at least 17 *Burkholderia* species, with *B. cenocepacia* being the one of the most common in CF (Drevinek and Mahenthiralingam, 2010). Other genera that have been previously described to be components of the CF airway microbiome include and were identified in our cohort include *Porphyromonas* spp., *Prevotella* spp., *Rothia* spp., *Streptococcus* spp., and *Veillonella* spp. (Harris et al., 2007; Tunney et al., 2008; Fodor et al., 2012; Zhao et al., 2012; Carmody et al., 2013; Zemanick et al., 2013; Lim et al., 2014). While PathoScope, Kraken, and MetaPhlAn all identified *B. cenocepacia* and *P. aeruginosa* as the dominant bacteria, lower abundance bacteria and the detection of viruses were not completely parallel. In addition, no bacteriophages were detected using PathoScope, but *Pseudomonas* phage B3 was detected using Kraken. *Propionibacterium* and *Staphylococcus* phages were also detected using MetaPhlAn. The limits in detection of bacteriophages in our samples are likely due in part to the smaller reference libraries for viruses and phages (Feigelman et al., 2017).

As PathoScope allowed for the detection of bacterial strain, it allowed us the opportunity to compare bacterial strains pre- and post-antibiotics. Interestingly, there was a shift in the relative abundance of a few strains of *P. aeruginosa* and *B. cenocepacia*. This might suggest that these strains possessed the necessary antibiotic resistance, while the other strains did not. Some prior studies demonstrated that *P. aeruginosa* decreased with antibiotic exposure during an acute pulmonary exacerbation (Zemanick et al., 2013). However, other studies have shown resilience of core bacteria within the CF airway microbiome with antibiotic use (Cuthbertson et al., 2016). Studies have microbial diversity following antibiotic use have also been mixed, with some showing decreased diversity (Zemanick et al., 2013; Smith et al., 2014), while other show no changes in diversity (Fodor et al., 2012; Price et al., 2013). The level of detail available using metagenomics and PathoScope could provide new insights into studies of individual bacterial abundance and microbial diversity of the CF airway in response to antibiotic use.

Using PathoScope, we were also able to evaluate the presence of fungal pathogens within the cystic fibrosis airway microbiome. *Candida albicans* and *Aspergillus fumigatus* are commonly detected in CF sputum cultures and have also been associated with acute pulmonary exacerbations (Willger et al., 2014). Sequencing studies of the CF lung mycobiome have also identified these pathogens. One study found that 74–99% of fungal reads were due to a mixture of *Candida* species and *Malassezia* (Willger et al., 2014). An earlier study found more diversity of fungal pathogens within four adult CF patients (Delhaes et al., 2012). In our study, we similarly identified the presence of several *Aspergillus* and *Candida* species. However, we also found more richness, with a total of 92 fungal species.

Our study has a few limitations. First, it is limited by the small number of subjects. Second, the contamination of human DNA in our sequencing may have affected our analysis. Our rates of 1–2% non-human reads are similar to other groups (Bacci et al., 2017). However, others have published that about a half a million reads are sufficient to provide a comprehensive metagenomic analysis of the taxa within the CF airway (Moran Losada et al., 2016).

## Conclusion

PathoScope outperformed Kraken and MetaPhlAn in our validation study of artificial bacterial community controls. PathoScope also has advantages over Kraken and MetaPhlAn in being able to determine bacterial strains and the presence of fungal organisms. Thus, PathoScope can be confidently used when evaluating metagenomic data to determine CF airway microbiome diversity.

## Availability of Data

The sequence data has been uploaded to NCBI under BioProject PRJNA422117.

## Ethics Statement

The study protocol was approved by the Institutional Review Board at Children’s National Health System and was carried out in accordance with their recommendations. All subjects gave written informed consent in accordance with the Declaration of Helsinki.

## Author Contributions

AH designed the study, performed the experiments, analyzed the data, and wrote the manuscript. MB contributed to study design, data analysis, and wrote sections of the manuscript. KG contributed to study design and data analysis. HC, IS, GP, and AK were all involved in study participant recruitment and sample collection. TM contributed to study design. RF and KC contributed to study design and interpretation of data analysis. All authors edited and approved the final manuscript.

## Conflict of Interest Statement

The authors declare that the research was conducted in the absence of any commercial or financial relationships that could be construed as a potential conflict of interest.
